# Non-polio enteroviruses among healthy children in the Philippines

**DOI:** 10.1186/s12889-020-8284-x

**Published:** 2020-02-03

**Authors:** Maria Melissa Ann Jiao, Lea Necitas Apostol, Maricel de Quiroz-Castro, Youngmee Jee, Vito Roque, Manuel Mapue, Frances Marsha Navarro, Cleo Fe Tabada, Amado Tandoc

**Affiliations:** 1 National Polio Laboratory, Department of Virology, Research Institute for Tropical Medicine, Muntinlupa City, Philippines; 2 World Health Organization Country Office, Manila, Philippines; 3 Center for Infectious Disease Research, National Institute of Health, Korea Center for Disease Control and Prevention, Cheongju, Chungcheongbuk-do South Korea; 4 Department of Health-Epidemiology Bureau, Manila, Philippines; 5 Department of Health-Center for Health Development NCR, Mandaluyong City, Philippines; 6 Department of Health-Center for Health Development Region VII, Cebu City, Philippines; 7 Department of Health-Center for Health Development Region XI, Davao City, Philippines

**Keywords:** Acute flaccid paralysis, Laboratory, Sequencing, Virus isolation, Coxsackievirus, Hand, Foot and mouth disease

## Abstract

**Background:**

Enteroviruses (EVs) are most commonly associated with either mild or asymptomatic infections, however, the presence of silent carriers in the community has been proven to play a crucial role in the spread of diseases such as hand, foot, and mouth disease (HFMD) that records high incidence in Asia Pacific region. In the Philippines, limited information is available on the etiology and prevalence of enterovirus outside the Acute Flaccid Paralysis (AFP) surveillance, thus, a study to determine the baseline prevalence of Non-Polio Enteroviruses (NPEVs) among healthy Filipino children was conducted.

**Methods:**

A descriptive, cross-sectional study was performed to determine the prevalence of NPEV among healthy children under 6 years old in the Philippines. Duplicate stool samples were collected from 360 healthy children residing in three major urban cities in the country. Virus isolation and polymerase chain reaction were performed to identify enteroviruses present in the samples. To determine if the results of the study are comparable to the AFP surveillance data, the results of the study were compared to the prevalence and isolation rate among AFP cases of the similar cases collected the same year.

**Results:**

Prevalence of enteroviruses among healthy children was found to be at 24.7%. Comparing the NPEV rates from the study and AFP surveillance of similar age and the same year of collection, there was no significant difference in NPEV case prevalence. The study identified a total of 19 different enterovirus serotypes with majority belonging to species Enterovirus B (EV-B).

**Conclusion:**

The study was able to establish a baseline NPEV case prevalence of 24.7% among healthy children aged under 6 years old in three major urban sites in the Philippines. The high isolation of NPEV among healthy children signifies continuous fecal-oral transmission of enteroviruses in the community.

## Background

Enteroviruses (EVs) of the *Picornaviridae* family are clustered into 15 species, of which, four are isolated exclusively in humans (EV- A to EV D) [1, 2]. EV infections are most commonly associated with either mild or asymptomatic infections. As an example, poliovirus, despite being highly contagious, has ratios of asymptomatic to paralytic cases that ranges from 50:1 to 1000:1 [3]. However, EVs are also associated with outbreaks of more serious diseases, such as hand, foot, and mouth disease (HFMD) and aseptic meningitis which results in considerable morbidity and mortality [4]. They are spread mainly through fecal-oral route with highest risk among children due to poor hygiene and low immunity levels.

It has been proven that asymptomatic carriers excreting EVs play a crucial role in the spread of poliovirus [5] and HFMD and their silent presences help perpetuate EV circulation in their community [6]. Evidence also suggests association between EV subtypes, particularly, Coxsackievirus B virus and chronic illnesses.

In the Philippines, the only information available about the epidemiology of EVs in the country is limited to cases reported under the Acute Flaccid Paralysis (AFP) surveillance [7]. The main importance of the isolation of EVs in AFP cases has been limited to its usefulness in assessing proper handling and transportation of AFP stool samples [8].

This study conducted in 2015 aimed to establish a baseline prevalence of non-polio enteroviruses (NPEVs) among healthy children under 6 years old in three selected sites in the Philippines and to identify the serotypes of the circulating EV in the community. The information gathered from this study will augment the information available on NPEVs in the country outside of what is captured by the AFP surveillance.

## Methods

### Study design

**Fig. 1:**
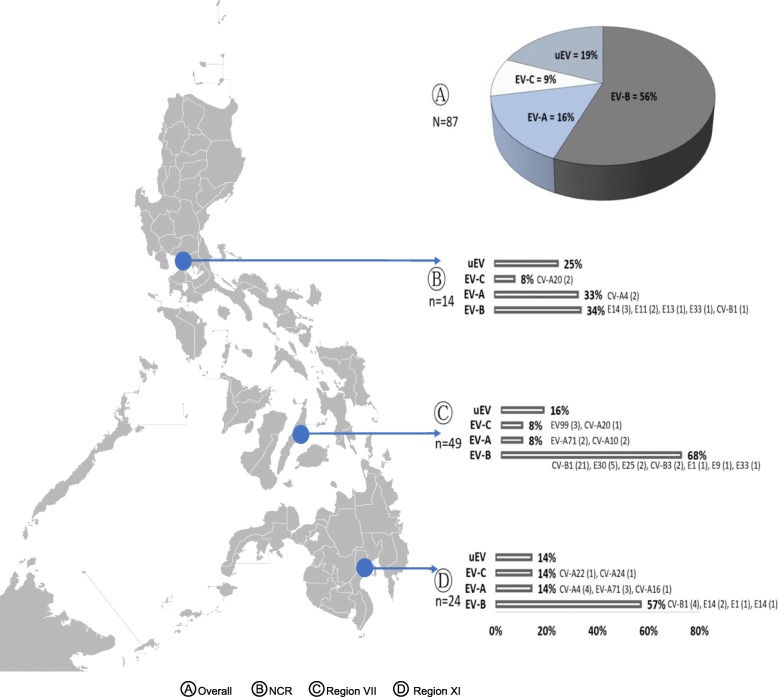
NPEV isolates among healthy children, Philippines, 2015. uEV (untyped EV) - CPE (+) samples on RD-A cell line and RNA detected in PCR but (-) in sequencing Image of Philippine map taken from https://commons.wikimedia.org/wiki/File:BlankMap-Philippines.png

A descriptive, cross-sectional study was designed to determine the prevalence of NPEV among healthy children under 6 years old in three major urban cities in the Philippines (Fig. 1). The study was conducted in urban areas in three different regions: (1) National Capital Region (NCR) in Barangay Addition Hills, Mandaluyong, Metro Manila; (2) Region VII in Barangay Carreta, Cebu City; and (3) Region XI in Barangay Buhangin, Davao City, Davao Philippines.

These sites were purposively selected as they were near the Department of Health-Regional Offices and had a barangay health center with sufficient cold storage equipment to store the collected stool samples. The minimum sample size computed for this study was 292. This was based on a 10.6% NPEV prevalence from a similar study conducted in China [9], 95% level of confidence, 5% precision estimate, design effect of 2. Additional 68 children were added to compensate for possible non-response, thus, a total of 360 healthy children (120 per site) under 6 years old were randomly selected from a sampling frame gathered through a survey of the health workers in their assignment areas. Only one child per household was permitted to join the study and the current health status of the participants was established to allow only healthy children to participate. “Healthy child” was defined as a child who, by clinical history and physical examination, did not present with symptoms that may be associated with enteroviral infections such as AFP, diarrhea, fever, cough, colds, conjunctivitis, and HFMD.

The study protocol was submitted to and approved by the Research Institute for Tropical Medicine (RITM)- Institutional Review Board and was conducted in compliance with the principles of the Declaration of Helsinki. Since the study participants are children under 6 years old, written informed consent was sought from the children’s parent or legal guardian. After the completion of viral testing, parents and guardians were informed of their ward’s results and those found to be positive for enteroviruses were advised to visit their health center physician for proper clinical management.

### Study procedures

#### Stool survey

The stool survey was performed between February and May 2015. Standard physical examination and clinical history were taken by study physicians to assess the child’s health status. Signs and symptoms that may indicate current enteroviral infection, such as AFP, gastroenteritis, influenza-like illness, encephalitis, myocarditis, HFMD, and conjunctivitis were evaluated. If the child was found healthy, the parents or guardians were instructed to collect two stool samples from their child, at least 24 h apart. Two stool samples were required to compensate for the intermittent shedding of the virus [10, 11]. To ensure that cold chain is maintained, parents and guardians were provided appropriate sample containers, ice pack, and a detailed instruction on proper sample collection. After collection, they were advised to forward samples to the barangay health center. From there, the samples were shipped by the Regional Epidemiology and Surveillance Unit staff to the National Reference Laboratory (NRL) for Polio and other Enteroviruses-RITM for testing.

#### Virus isolation

Stool samples were labeled with the study identification numbers prior to sample processing and testing. Virus isolation was performed at the NRL for Polio and other Enteroviruses-RITM, following the WHO standard procedures for poliovirus isolation. Briefly, all stool samples were treated with chloroform and antibiotics, then, 200 μL of the stool extracts was inoculated into two rhabdomyosarcoma (RD-A) and two L20B cell lines. EVs are very diverse; thus, no single cell line is susceptible to all EV species. L20B is specific for polioviruses while RD-A can grow most EV species. These tubes were observed for a total of 10–14 days and the infected tissue culture fluids (ITCF) of tubes showing the characteristic cytopathic effect (CPE) of enterovirus (rounding necrosis) were harvested and stored for subsequent testing [8]. Negative cell control tubes were also observed together with the samples and these were used to compare the appearance of the normal, healthy cells and the infected ones.

#### Sequencing

EV-positive ITCFs were selected and sent to the National Institute for Health- Korea Center for Disease Control and Prevention (NIH-KCDC) for partial VP1 gene sequencing according to the institute’s protocol. VP1 was targeted since it is the best region for phylogeny-based classification. If case both stool samples of the child yielded NPEVs, only the first stool isolate was included for sequencing.

In brief, viral ribonucleic acid (RNA) was extracted using Tecan Freedom Evo™ (Tecan Group Ltd., Männedorf, Switzerland). Enterovirus gene was amplified through polymerase chain reaction (PCR) using iNtRON iNNOPLEX™ Enterovirus VP1 detection kit (Bulldog Bio, Inc., Portsmouth, New Hampshire). Amplified products were then sent to Macrogen Korea and to Cosmogenetech Korea for Sanger sequencing. Sequencing results were cleaned and aligned using DNA Star™ software (DNASTAR, Inc., Madison, Wisconsin) and MEGA software v.7 A BLAST search was then conducted to identify the enterovirus serotype [12]. Sequences generated from the study were submitted to GenBank with accession numbers: MK959771 to MK959836 and MK977636 to MK977640.

### Statistical analysis

All questionnaires and signed informed consent forms were checked in the field for completeness. Data entry was performed using Epi Info v. 3.5.3 (Centers for Disease Control and Prevention, Atlanta, Georgia). Comparison of sex, age group and location with the NPEV prevalence as well as comparison in between groups was analyzed by using Chi-square test, and *p*-values < 0.05 were considered statistically significant. The data gathered from the study were also compared to AFP surveillance cases under 6 years old.

## Results

### Prevalence of NPEV

The mean age of the participants is 2.4 and an almost equal proportion of males and females, 51 and 49%, respectively.

Two stool samples were collected from each participant. Of the 720 total collected stool samples from 360 children, NPEVs were isolated in 129 (17.9%) samples from 89 cases. Of these 129 samples, 126 were single isolates of EVs while three were mixed with poliovirus type 3, Sabin-like strain. NPEVs were isolated in 89 children (89/360) or 24.7% of participants. Among these positive participants, 50.6% (45/89) are females and 60.7% (54/89) belong to the 1 to 3 years old age group. Among the three study sites, Region VII had the highest detected case prevalence with 51 out of the 120 study participants (42.5%) yielded at least 1 NPEV from their stool samples. NPEV case prevalence was different among the study sites (χ^2^ = 32.81, *p*-value = < 0.001, df = 2). Likewise, the NPEV case prevalence in Region VII was highest followed by NCR and Region XI, respectively. Analysis showed also that there was no significant difference in NPEV case prevalence among males and females and also between age groups in the study (Table 1).

**Table 1:** Demographic information of participants during 2015

Characteristics	NPEV Positive		NPEV Negative		χ^2^ test	*p*-value
	No.	%	No.	%		
Sex						
Males	44	23.78	141	76.22	0.09	0.763
Females	45	25.71	130	74.29		
Age Group						
< 1 year	13	21.67	47	78.33	3.71	0.156
1–3 years	54	28.88	133	71.12		
4–5 years	22	19.47	91	80.53		
Geographic Location						
NCR	24	20.00	96	80.00	32.81	<0.001
Region VII	51	42.50	69	57.50		
Region XI	14	11.67	106	88.33		

Comparative analysis on the NPEV case prevalence and isolation rate (Table 2) reported by the study with those reported AFP cases under 6 years old in 2015 was also done. There was no significant difference in both NPEV case prevalence and NPEV isolation rate except for Region XI where there was a difference in terms of NPEV isolation rate among cases (*p*-value 0.046) (Table 2).

**Table 2:** Comparison of NPEV case prevalence and isolation rate among healthy children and AFP cases under 6 old, 2015

Isolation	NPEV Positive		NPEV Negative		χ^2^ test	*p*-value	Case	NPEV Positive		NPEV Negative		χ^2^ test	*p*-value
	No.	%	No.	%				No.	%	No.	%		
NCR													
HC AFP < 6	20 4	8.3 7.8	220 47	91.7 92.2		*0.59	HC AFP < 6	14 2	11.7 7.4	106 25	88.3 92.6		*0.40
Region VII													
HC AFP < 6	77 2	32.1 14.3	163 12	67.9 85.7		0.010	HC AFP < 6	51 2	42.5 28.6	69 5	57.5 71.4		*0.38
Region XI													
HC AFP < 6	32 10	13.3 25.6	208 29	86.7 74.4	3.97	0.84	HC AFP < 6	24 6	20.0 31.6	96 13	80.0 68.4		*0.20
All sites/ Philippines													
HC AFP < 6	129 51	17.9 16.9	591 251	82.1 83.1	0.16	< 0.001	HC AFP < 6	89 32	24.7 20.8	271 122	75.3 79.2	0.93	0.334

*Fisher exact test *p*-value

### Molecular sequencing result

The study identified a total of 19 different enterovirus serotypes with majority belonging to species EV-B with 11 different serotypes detected. The predominant circulation pattern of the EV-B species was seen in all sites – NCR at 57%, Region VII at 63% and Region XI at 34%. No subtype or serotype under EV-D was detected. Isolation from EV-A (16%) was higher than EV-C (9%). Coxsackievirus B1 (CV-B1) is the most common as it comprised 29.9% of the NPEVs identified. In this study, due to the limitation of the method used, only 81% of the NPEVs isolated were characterized. The remaining 19% of NPEVs did not produce clean sequences and thus, were termed as untypable EVs (uEVs).

## Discussion

The study aims to determine the baseline NPEV rate among healthy Filipino children under 6 years old. In this study, the prevalence among participants is 24.7%, as NPEVs were isolated in the stool sample of 89 out of 360 enrolled participants. In contrast with the studies done in Indonesia and South-Western India [13, 14], the notion that males are more likely to contract EV infection was not established in this study. The study revealed that NPEV case prevalence among these age brackets (<1, 1–3 and 4–5) were almost equal indicating that the chances of NPEV infection among these age groups, from infancy to preschool age, is comparable.

The study was able to establish the predominant circulation EV-B species, particularly, CV-B1 which accounted for around 30% of all EV species isolated. The isolation of CV-B1 in all sites is consistent with a previous study where Coxsackievirus B (CVB) was classified as one of the EV serotypes with endemic circulation in the Philippines [15]. This EV species has been known to be the most common viral cause of human heart infections [16]. There was also an isolation of CV-B4 from a child enrolled in the study and this subtype is known to be an environmental risk factor in the non-genetic causes of type 1 diabetes mellitus [17].

Previous study on the characterization of enterovirus isolates from AFP cases in the Philippines showed that the circulating isolates are, in decreasing order, EV-B, followed by EV-C then by EV-A species [7]. This circulation pattern is also seen in other studies from Asian countries [18, 19]. This pattern was in contrast with the results of this study wherein a minor difference in the proportion was seen for EV-A and EV-C species. While all sites followed the isolation pattern of EV-B > EV-A > EV-C, there is a variation in the proportion of species detected per region especially in NCR where EV-A has almost the same as EV-B isolation. All of the detected EV serotypes among healthy children mirror the EV serotypes isolated from AFP cases and environmental samples in previous reports except for the detection of CV-A22. The detection of CV-A22 in the Philippines was first identified and documented in this study [7, 20].

The study was also able to detect EV-A71 and CV-A16 in healthy children and this finding is significant as these are usually associated with HFMD [21–23]. This finding, however, is not exclusive to this study, as a similar result was seen in the survey of healthy children conducted in Shenzhen and Yunnan Province, China. The isolation of these pathogens among healthy population is considered an important factor in the continuous circulation of the disease in the community [6, 9]. These EV types were also detected in previous studies on NPEVs among AFP cases and environmental samples in the Philippines, which may be suggestive of indigenous circulation of these pathogens in the country [7, 20]. Further analysis indicated that the EV-A71 in this study belonged to genogroup C, specifically the C2 cluster which is genetically homologous to the EV-A71 C2 cluster reported among AFP cases [15]. While neighboring countries in the Asia Pacific region revealed high mortality rates caused by EV-A71 of the genogroup C such as in China, [24] the Philippines has yet to report any fatal case. Data from a comprehensive and longitudinal HFMD study on the overall EV-A71 epidemiology is crucial to conclude that C2 cluster of EV-A71 found in the Philippines is only causing mild disease or even asymptomatic infection.

The age range of all AFP cases is children under 15 years old, therefore, the study decided to compare the results of the study from AFP cases of the same age because a study conducted in Sweden from 2003 to 2007 has shown that the peak of EV isolation is among 18 month-old participants [25] and several other studies suggest that enteroviral infection is greater among younger children [13, 26, 27]. The fact that the study participants’ ages (0–5 years old) do not match those of the AFP surveillance cases (0–15 years old) might be a factor for the difference in NPEV case prevalence and isolation rate.

## Conclusion

The study was able to establish a baseline NPEV case prevalence of 24.7% among healthy children aged under 6 years old in three major urban sites in the Philippines. The high isolation of NPEV among healthy children signifies continuous fecal-oral transmission. The study was also able to determine EV-B as the most prevalent species of EV in the country.

### Limitation of the study

The study accepts the limitation brought about by using only two cell lines to detect EVs. Several studies conducted showed that the best cell line to use is the MRC5 cells as it yields the best result [28, 29]. The laboratory elected to use the RD-A and L20B cell lines and follow the WHO protocol for poliovirus identification. Despite this, the study was able to isolate CV-A22. CV-A22 was first isolated from a healthy person from Chulman, Russia [30] and this type is rarely seen because of its difficulty to be isolated through tissue culture techniques [31]. In this study, the isolate came from a 3 years old child from Region XI.

The molecular method used in the study limited the detection of EVs as uEVs accounts for 19% of the overall EV isolation. The gene targeted by the assay is the VP1 region since it is the best region for phylogeny based classification [32, 33]. For a more comprehensive picture on diversity of EVs among healthy children, a more sensitive method that could identify all possible EV serotypes should be used for subsequent studies. Nonetheless, the method was able to detect newer EVs like Enterovirus C99 (EV-C99).

## Data Availability

The datasets generated and analyzed during the study are not publicly available for the data privacy protection of participants but are available from the corresponding author on reasonable request. The partial VP1 sequences of the enterovirus were deposited in GenBank under accession numbers: MK959771 to MK959836 and MK977636 to MK977640.
